# Evidence-based practice in well-child care

**DOI:** 10.1007/s00431-022-04624-3

**Published:** 2022-09-28

**Authors:** JJ De Schipper, AJM Hermans, ADC Jaarsma, FW Noordik, SA Reijneveld

**Affiliations:** 1 Netherlands School of Public and Occupational Health, Utrecht, The Netherlands; 2 NSPOH C/O JJ de Schipper, Postbox 20022, Utrecht, 3502 LA The Netherlands; 3 Department of Health Sciences, University Medical Center, University of Groningen, Groningen, The Netherlands; 4 Faculty of Veterinary Medicine, Utrecht University, Utrecht, The Netherlands

**Keywords:** Well-child care, Community pediatricians, Evidence-based practice, Evidence-based medicine, Postgraduate medical education

## Abstract

Evidence-based practice (EBP) significantly improves the quality of healthcare, but its use in community pediatrics has not yet been proven. We aimed to assess how Dutch community pediatricians use scientific findings and apply evidence-based practice in everyday well-child care. We interviewed a purposive sample of 14 community pediatricians in the Netherlands regarding their professional activities in daily practice, focusing on instances in which their professional knowledge was insufficient to address the issue at hand. We transcribed the interviews verbatim, and coded them using ATLAS.ti software. We structured the information using template analysis. Community pediatricians relied largely on guidelines of their own profession. If these were not sufficient, they first consulted other medical specialists or colleagues, or used different sources that they considered reliable. They only rarely performed an EBP search, and if so, only for somatic problems. For psychosocial problems, they used a strategy of extensive interaction with clients and members of multidisciplinary teams. We identified five barriers to performing an EBP search: (1) a conviction that not every community pediatrician needs to be able to perform an EBP search; (2) a conviction that an EBP search is not suitable for psychosocial problems; (3) lack of confidence in one’s own abilities to perform an EBP search; (4) limited access to literature; (5) lack of time.

*Conclusions*: Community pediatricians rely on professional guidelines; this indicates a need to keep these up-to-date and user-friendly. Furthermore, pediatricians should be better trained in performing EBP searches, and in working in multidisciplinary teams, especially for psychosocial problems.

**Table:** 

**What is Known:**
• *Conducting an evidence-based practice search is considered indispensable to determine the best management of the patient’s problem.*
• *Conducting such a search is still considered challenging in many medical disciplines, including pediatrics.*
**What is New:**
• *There is a need to strengthen skills of community pediatricians to find evidence on psychosocial problems and to present this effectively in multidisciplinary teams.*
• *The pediatricians’ broad use of other sources of evidence, like experts and online sources, shows the importance of critical evaluation skills.*

**Supplementary Information:**

The online version contains supplementary material available at 10.1007/s00431-022-04624-3.

## Introduction

Evidence-based medicine has a broadly supported role in improving the quality of healthcare [1, 2], but evidence is lacking regarding the extent of its use in community pediatrics. Kelly [1] has described the work of researchers like Sackett and Cochrane to establish the value of evidence-based medicine. Furthermore, Sackett et al. [3] have affirmed the need to combine evidence with clinical expertise and patients’ choices when making medical decisions. Finally, Dawes et al. [4] have proposed the broader concept known as evidence-based practice (EBP). Using this concept, we also define an EBP search as a process involving five steps: ask, access, assess, apply, and audit. Many medical disciplines have developed evidence-based guidelines, with state-of-the-art recommendations for common patient problems, to support EBP. When guidelines are inadequate or outdated, an EBP search is considered indispensable to determine the best management of the patient’s problem. However, conducting such a search is still considered challenging in many medical disciplines [5–7], including pediatrics [8].

Regarding pediatric practice, Draaisma et al. [8] have pointed out that, although for pediatricians in clinical care it was difficult to conduct an EBP search regularly, this could be promoted with the use of proper interventions. Whether that also holds for community pediatricians is unknown. The Dutch setting provides an excellent opportunity to assess this application of EBP in community pediatrics, as in the Netherlands well-child care is provided by community pediatricians who do not provide clinical care. Therefore, the aim of this study was to assess how Dutch community pediatricians use scientific evidence and how they apply EBP principles in everyday well-child care.

## Methods

For this study, we used semi-structured interviews with community pediatricians.

### Sample

We obtained a purposive sample of 14 participants from among 96 community pediatricians who graduated between January 2015 and January 2018 from the Netherlands School of Public and Occupational Health (NSPOH), one of the two Dutch postgraduate medical schools for community pediatricians. Roughly 60% of community pediatricians in the Netherlands are trained at the NSPOH. To maximize variability, we selected participants from different work situations (employed in a well-child clinic or a municipal public health service) and with varied education (two-years training as community pediatrician or 4-year training as community medicine specialist in pediatric care).

**Fig. 1:**
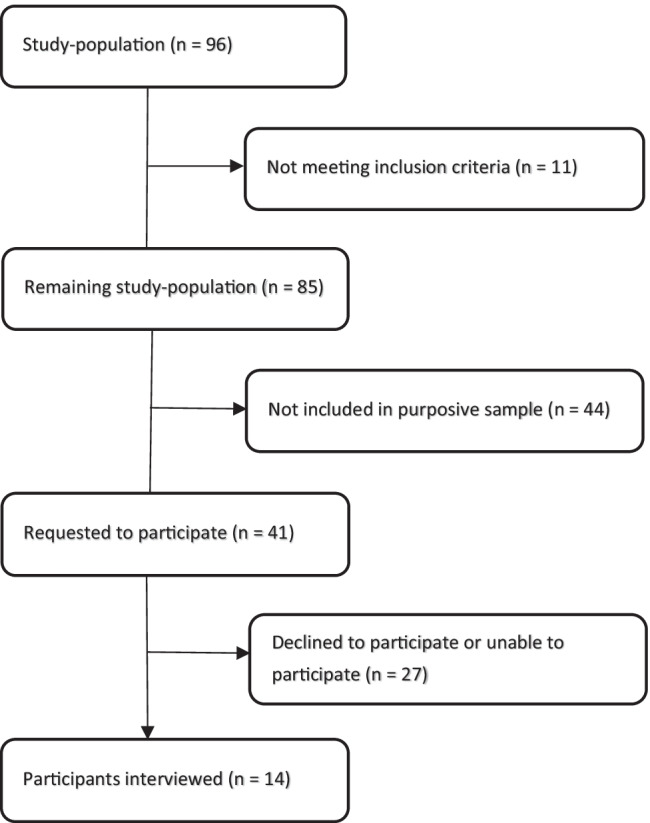
Flow diagram of participant selection

Participants were invited by e-mail and, in case of no reply, by telephone. They were eligible if they were actually working as a community pediatrician in daily practice, and not involved in further training as a general specialist in community medicine. Figure 1 shows the flow of participant selection. The Ethical Review Board of the Netherlands Association for Medical Education approved the study protocol (file 2018.7.6).

### Procedure and measures

Each participant received a questionnaire (Supplementary File 1) on background characteristics, and was interviewed by the first author JJdS between February 2019 and August 2020. JJdS is an educational scientist, trained in interviewing techniques. He was responsible for organizing the training on EBP principles for the participants at the NSPOH. All participants received information about the study, including its aims and content; each signed an informed-consent form.

Interviews were conducted in a semi-structured way, using a topic-list; they lasted about one hour, and were audio-recorded. Five participants were interviewed face-to-face, the other nine by phone, in three cases because of COVID-related restraints. The topic-list was tested in a pilot interview, resulting in the following lead question in the final version: “If you have to make a medical decision or provide a client with advice, but your professional knowledge is not sufficient, how do you deal with that? Could you please provide some examples?” The interviewer subsequently focused on what the participant did to gain enough basis for a decision or an advice. If topics like use of evidence, clinical expertise, and patients’ choice, according to categories proposed by Sackett [3], or specifics regarding the steps of an EBP search [4] were not mentioned spontaneously, the interviewer asked specific questions about them. Follow-up questions focused on the attitude of colleagues and the organization [5] toward EBP. After the fourth interview we added a question about the obligatory peer-review sessions for community pediatricians, because in that interview these sessions appeared to be a useful context for an EBP search. The final version of the topic list is shown in Supplementary File 2.

### Data analysis

All interviews were transcribed verbatim by a certified external office and coded in ATLAS.ti by JJdS. Data analysis was performed simultaneously with the interviews, in line with the principles of template analysis [9]. To improve information power, we continued to conduct interviews until no new codes appeared from the data. Four interviews were double coded, two by AJMH, a community medicine specialist in pediatric care, and two by FWN, a trainer in basics of research methodology. The code-tree was discussed repeatedly with all authors and adjusted cumulatively during the coding; its final version is shown in Supplementary File 3.

## Results

### Characteristics of the sample

Participants worked at twelve different organizations across the Netherlands. Their mean age was 48 years (SD 7.3; range 33–60). Thirteen were female and one male. They had completed their undergraduate medical education 10 to 22 years previous to the interview. Two participants had followed an extra course on EBP and two had contributed to a publication on community pediatrics. Ten had followed a 2 years’ basic specialist training, and four the 4 years’ specialist training.

### How do community pediatricians in well-child care use scientific evidence?

Most participants considered it important to keep their professional knowledge up to date. If their knowledge appeared inadequate in a specific professional context with a client, they reported handling this first by consulting professional guidelines and other medical specialists. If that did not suffice, they used varied follow-up approaches for somatic and for psychosocial problems, as outlined further.

#### Keep professional knowledge up to date

As important reasons to keep their knowledge up to date, participants mentioned the ability to provide adequate care and to confer effectively with other disciplines. They kept track of new developments in their area of expertise by consulting a limited number of scientific journals, training programs, and newsletters, but often also social media: [29^1^: “I read … a lot of professional information on all kinds of topics that are also shared via Twitter, for example, and … then I read the original research”].

Most participants considered indirect access to literature adequate for this purpose. However, lack of time often prevented them from keeping their knowledge up to date: reading professional literature was often a spare time activity.

#### In case of gaps in professional knowledge: consult professional guidelines, peers, or other specialists

When in doubt, participants first checked the guidelines of the Dutch national expertise center on well-child care, the NCJ [10]. These evidence-based professional guidelines provide guidance for a considerable number of somatic and psychosocial problems. They were broadly used and appreciated: [15: “Well, just look at all those guidelines, they really help us in our work. And how did they come about? By research!”]. Sometimes, no relevant guideline was available, or an existing guideline was outdated. In such cases, the participants conferred with colleagues or with other medical specialists, who could easily be reached for a telephone or digital consultation. Participants also used other sources considered to be reliable, like websites from pediatricians and dermatologists or relevant guidelines from other disciplines.

#### Different follow-up approaches for somatic or psychosocial problems

For somatic problems, the community pediatricians generally could decide independently on the course of action. The consultation stage mentioned above usually provided them with sufficient information. If not, some participants searched for specific studies or started a systematic EBP search. Such searches were performed sometimes individually, and sometimes with other colleagues, as part of peer-review sessions.

However, for psychosocial problems, the participants indicated that guidelines were not always available, and if so, they were often not sufficient to rely on for decisions or advice. The complexity of the clients’ problems usually led to a different consultation stage, characterized by extensive interaction with clients: [15: “…for a behavioral problem, it is more like practicing judo with parents … what is their concern, what is mine? … It becomes more practice-based”]. Also, not the individual community pediatrician, but members of multidisciplinary teams, consisting of nurses, social workers, and psychologists, decided on the course of action. Working in multidisciplinary teams was sometimes very effective, but could cause frustration as well: [26: “… They do not listen to evidence … We are all considered equal, a doctor with more than 20 years of experience and an advisor who has just started working … and then the person who submits the case … can choose which advice he wants to follow”]. For psychosocial problems, none of the participants considered starting an EBP search useful.

### How do community pediatricians in well-child care apply EBP principles?

Regarding application of EBP principles, most participants indicated that in daily practice they never conducted a formal EBP search; the few examples mentioned are described below. Our study identified five barriers to performing an EBP search and these too are described below.

#### Examples of EBP searches performed

Some participants had performed partial EBP searches to find up-to-date answers on specific questions of clients: [43: “Sometimes parents … have a very clear opinion on weaning. …. Then I also look up … the latest studies on breastfeeding and supplementary feeding”]. Other EBP searches were aimed at developing organization-specific evidence-based guidelines for common practical questions. An interesting example was the use by one organization of peer-review sessions to perform EBP searches together: [25: “We … recently discussed … micro- and macrocephaly … so that we can make … better working agreements for colleagues; we … will, … look up articles about it … and make a summary and discuss it … during the peer-review session”].

#### Barriers mentioned

Participants mentioned the following five barriers to using an EBP search:

Barrier 1: Conviction that not every community pediatrician needs to be able to perform an EBP search

Some participants strongly indicated that being able to perform an EBP search is not necessary to perform well as a community pediatrician: [36: “I’m more for practice-based evidence than evidence-based practice. We can’t all be scientists…. Let me just examine the kids and give guidance to the parents—I’m good at that”]. Other participants stressed the opposite, namely that they considered it important for every community pediatrician to be able to derive substantiated advice from relevant literature.

Barrier 2: Conviction that an EBP search is not suitable for psychosocial problems

The participants considered it obvious that an EBP search is most appropriate in relation to somatic questions. They indicated that a search in PubMed was not likely to be successful for psychosocial problems: [15: “… breastfeeding and a certain medication is specific and small and medical; PubMed is suitable for this; … if you … start looking for social stuff in PubMed you will get bogged down very quickly”]. If they did mention using peer-review sessions to find answers to psychosocial problems, these sessions were aimed at discussing cases and not at performing an EBP search in order to support a decision or give advice.

Barrier 3: Lack of confidence in one’s own abilities to perform an EBP search

Some participants indicated feeling sufficiently confident to conduct an EBP search. However, most felt inadequate to do so, especially in comparison with colleagues who had much more recently completed their undergraduate medical education*.*

Barrier 4: Limited access to literature

Those participants who conducted EBP searches had access to full-text literature through a shared license. Of those who did not, some mentioned that they had to request specific literature from a library; as a result, this was a barrier to using literature: [36: “… if scientific literature would be much easier to access … one might be more inclined to look for it”].

Barrier 5: Lack of time

Most participants mentioned lack of time as a hindrance to gaining the experience needed to do a thorough EBP search: [16: “… If you don’t do that very often, it takes a lot more time and you don’t always allow yourself that time”].

## Discussion

We found that community pediatricians first preferred to consult the national guidelines of their own profession when their knowledge was insufficient to give advice or make decisions. Subsequently, they preferred to consult other experts and sources they considered reliable. For somatic problems, they usually found this procedure to be sufficient as a base for their advice or decisions. They occasionally performed additional EBP searches. However, for psychosocial problems, it was necessary to have much more interaction with clients and within multidisciplinary teams, and they never conducted EBP searches.

We found five barriers that prevented community pediatricians from performing an EBP search: a conviction that not every community pediatrician needs to be able to perform an EBP search; a conviction that an EBP search is not suitable for psychosocial problems; lack of confidence in one’s own abilities to perform an EBP search; limited access to literature; and lack of time.

### Interpretation of main findings

We found that the community pediatricians preferred national professional guidelines, and only subsequently applied a broader strategy to find evidence. This aligns with the finding that the 35 available guidelines provide adequate evidence and cover the routine offer of well-child care [11], which is important in professional situations when time is scarce. We found that for psychosocial problems, the community pediatricians moved quickly from the use of guidelines to a strategy of intensive interaction with clients and multidisciplinary teams; a possible explanation is that they considered these guidelines less applicable to the complex problems of their clients. We also found that working in multi-disciplinary teams posed an extra challenge — for instance, to reach consensus about the client’s problem and to acknowledge the contribution and value of each discipline involved. This finding aligns with that of Dopson et al. [12] that different professions can have different views about what constitutes credible evidence.

We found that the community pediatricians seldom performed an EBP search. This finding aligns with findings related to other disciplines [5–7], although we found some different barriers. The first barrier — the conviction that not every community pediatrician needs to be able to perform an EBP search — contrasts with the general support for EBP in all medical professions [1, 2], including AJN Jeugdartsen Nederland, the Dutch organization of community pediatricians [13]. The second barrier — the conviction that an EBP search is not considered suitable for psychosocial problems — is a remarkable finding. It contrasts with the fact that there is published evidence on the best handling of psychosocial problems (e.g. [14–17]). This incorrect assumption prevents pediatricians from making use of existing evidence. The third barrier — lack of confidence in one’s own abilities to perform an EBP search — is possibly caused by a lack of practice in using EBP. The participants graduated from their professional medical education 10 to 20 years ago, at a time when intensive training in using EBP principles was less common and the online EBP search possibilities were limited and more complicated. The fourth barrier — limited and incomplete access to literature — and the fifth barrier — lack of time — both align with findings in other studies [5, 18, 19].

In summary, the community pediatricians, like other medical specialists, reported that they valued professional guidelines as an important source of evidence, and they seldom performed EBP searches. For dealing with psychosocial problems, they considered it obvious that EBP searches would not be successful, a conclusion which contrasts with the availability of published evidence.

### Strengths and limitations

A strength of this study is that it yielded a rich description of the results, with illustrative citations, in an area where evidence was not yet available. Another strength is the diversity of the research team, made up of both community pediatricians and educational scientists. This provided a multi-perspective interpretation of the data, thereby augmenting the credibility of the results.

A limitation of the study is that participants included graduates from only one of the two schools for community pediatricians in the Netherlands. However, the schools’ curriculums on EBP are broadly comparable, implying that our findings most likely apply to graduates from both schools. Another limitation may be that interviewer JJdS was also involved in the EBP training of the participants. However, that some answers were socially less desirable indicates that the interview setting was probably considered safe. A third possible limitation is that we interviewed a limited group, of only 14 participants. However, our sample of the study population was purposeful, resulting in a sufficiently heterogeneous group of participants. Also, the final three interviews which we used for verification of findings yielded no new information or codes. This suggests the presence of sufficient information power.

### Implications and conclusion

Community pediatricians used national professional guidelines as their main source of scientific evidence; this indicates the value of these guidelines as well as the need for regular and frequent updates and extensions. Community pediatricians need more training and practice in skills to perform EBP searches and need sufficient time and unlimited access to literature. The pediatricians’ broad use of other sources of evidence, like experts and online sources, shows the importance of critical evaluation skills. Regarding the relatively new development of working in multi-disciplinary teams for psychosocial cases, our findings suggest a need for training in how to grade the quality of the evidence found in literature databases and other sources and how to communicate this information effectively within interdisciplinary settings. Our findings regarding barriers indicate a need to strengthen practitioners’ skills to find evidence on psychosocial problems. We identified the conducting of a systematic EBP search with colleagues in peer-review sessions as a best practice, and recommend that community pediatricians in more organizations begin to implement this practice. In conclusion, we found that in their everyday well-child care, most Dutch community pediatricians relied for evidence first on national guidelines and then on other sources, but they seldom performed an EBP search. Implications for practice are that excellent and up-to-date national guidelines are important and that several important skills should receive attention during postgraduate education, preferably combined with practice in the work setting: skills to perform EBP searches, critical evaluation skills, skills to work in multidisciplinary teams, and skills to find evidence on psychosocial problems. Our findings need confirmation in other countries with different systems of pediatric care.

## Supplementary Information

Below is the link to the electronic supplementary material.

Supplementary file1 (DOCX 20 KB)

## Abbreviations

EBP: Evidence-based practice
NCJ: Dutch national expertise center on well-child care — Nederlands Centrum Jeugdgezondheid
NSPOH: Netherlands School of Public and Occupational Health

## Footnotes

1 Quotes of participants are given between brackets and in italics, and are preceded by a unique (Fig. 1) participant number.
