# Supplementary material for: Evidence-based practice in well-child care
Source: Eur J Pediatr. 2022 Sep 28;181(12):4183–9. doi: 10.1007/s00431-022-04624-3 (PMC9649461; doi:10.1007/s00431-022-04624-3)
Supplement: Supplementary file 1 — Supplementary file1 (DOCX 20 KB) [file 431_2022_4624_MOESM1_ESM.docx]

# SUPPLEMENTARY MATERIALS

## Questionnaire

Please complete prior to the interview and send to the address below.

1. Personal data:
   1. Sex: M / F (please circle what applies)
   2. Age: ……….….. years old
2. Medical education:
   1. I completed my medical studies in …………. (year)
   2. I am a community pediatrician (2-years training), registered in …..… (year)
   3. I am a community medicine specialist in pediatric care (4 years training)
      1. yes / no
      2. if yes, registered in …………….. (year)
3. I have followed specific Evidence-Based Medicine training

in addition to the aforementioned education yes / no

1. I have contributed to a publication in a scientific journal yes / no
2. Employer (please circle what applies; more options are possible):

municipal public health service / well-child clinic / other: ………………..

1. Function: ……………………………………………………………………………………………………………………
2. Main tasks in the work situation:

- ……………………………………………………………………………………………………………….
- ……………………………………………………………………………………………………………….
- ……………………………………………………………………………………………………………….

Please fill out and send to ………………………………………………. (please mention: research, confidential)

interview number ……. (to be completed by the researcher)

## Topic list for the interview

A. Introduction: welcome, briefly explain the purpose of the study and the process of the interview, and check whether participant has any questions before beginning the interview.

NB There are no right or wrong answers; I am particularly curious about whether and how you seek and find substantiation for your advice and decisions during your consultation hours.

B. View the completed questionnaire and, if necessary, request further explanation

C. Questions:

- To get to know your work a little better: can you tell me a bit more about your work and the clients you see?
- Please describe an average day for me: what kinds of clients and cases do you encounter? What kinds of activities / tasks do you do?
- What kinds of medical decisions do you make? What kinds of advice do you give? Please give some examples.
- “If you have to make a medical decision or provide a client with advice, but your professional knowledge is not sufficient, how do you deal with that? Could you please provide some examples?”
- If not, keep asking questions: what do you base your decisions and advice on?
- If yes, ask for specific examples:
  - What exactly did you not know? What exactly were you uncertain about?
  - What did you do to find the actual information - to remove your doubts or reduce your uncertainty?
  - Did that help you? Why?
  - On what basis did you ultimately make a decision or give advice?

Possible in-depth questions (if the relevant topics have not yet been discussed):

- To what extent did you use your own experience to substantiate your decision?
- To what extent did you use patient preferences and values to support your decision?
- To what extent did you use scientific evidence to support your decision?
  - If you were looking for scientific information at the time, how did you proceed? Camera perspective…
  - What did you do well? What made this go well?
  - What did you do less well? How did that happen? How did you solve those difficulties? What else would help you to overcome those difficulties?
- Possible additional general questions (unless the relevant topics have already been discussed in the examples):
- How do your colleagues deal with EBP when it comes to 'consultation hour issues'?
- How does the/your organization deal with EBP? Can you explain that? What does that indicate?
- Describe the formally required peer review sessions in your organization. (Question added after 4^th^ interview)

## Code tree

1. General:
2. Keeping updated in own discipline, passively or actively

- individual assessment of importance of keeping updated
- access to literature
- time to read literature

1. Usually, sufficient available professional knowledge
2. In case of insufficient available professional knowledge with respect to somatic problems:
3. Used initially

- NCJ Guidelines

1. Used in second instance if NCJ guideline nonexistent or insufficient:

- consultation with colleagues or other specialists
- reliable other online sources

1. Guidelines, conferring, and other sources usually sufficient support to make a decision or give advice. If not:

- sometimes individual EBP-search
- sometimes joint EBP-search during peer review sessions

1. In case of insufficient available professional knowledge with respect to psychosocial (multi-) problems:
2. The abovementioned actions (2a and 2b) provide insufficient support to make decisions or give advice.
3. This causes a long and less clear-cut path to decisions or advice:

- use of case study consultation with colleagues
- deployment of multidisciplinary teams
- EBP not considered a feasible option

1. Factors influencing an EBP-search
2. Context

- goal: individual questions or organizational guidelines
- actor: individual professional, or group of professionals

1. Possible barriers

- value: community pediatrician’s opinion regarding importance of EBP-skills
- difference in type of problem: somatic versus psychosocial
- self-evaluation: low estimation of skills needed
- literature: difficult access to literature
- time: lack of time to carry out EBP
